# Oxytocin Normalizes Approach–Avoidance Behavior in Women With Borderline Personality Disorder

**DOI:** 10.3389/fpsyt.2020.00120

**Published:** 2020-03-11

**Authors:** Isabella Schneider, Sabrina Boll, Inge Volman, Karin Roelofs, Angelika Spohn, Sabine C. Herpertz, Katja Bertsch

**Affiliations:** ^1^Department of General Psychiatry, Center of Psychosocial Medicine, University of Heidelberg, Heidelberg, Germany; ^2^FMRIB Centre University of Oxford, Nuffield Department of Clinical Neurosciences, John Radcliffe Hospital, Oxford, United Kingdom; ^3^Behavioral Science Institute, Radboud University, Nijmegen, Netherlands; ^4^Department of Psychosomatic Medicine, Central Institute of Mental Health Mannheim, Medical Faculty Mannheim/Heidelberg University, Mannheim, Germany; ^5^Department of Psychology, LMU Munich, Munich, Germany

**Keywords:** placebo, reaction time, angry, happy, congruency effect

## Abstract

**Background:** Interpersonal deficits are a core symptom of borderline personality disorder (BPD), which could be related to increased social threat sensitivity and a tendency to approach rather than avoid interpersonal threats. The neuropeptide oxytocin has been shown to reduce threat sensitivity in patients with BPD and to modify approach–avoidance behavior in healthy volunteers.

**Methods:** In a randomized, double-blind placebo-controlled between-subject design, 53 unmedicated women with BPD and 61 healthy women participated in an approach–avoidance task 75 min after intranasal substance administration (24 IU of oxytocin or placebo). The task assesses automatic approach–avoidance tendencies in reaction to facial expressions of happiness and anger.

**Results:** While healthy participants responded faster to happy than angry faces, the opposite response pattern, that is, faster reactions to angry than happy faces, was found in patients with BPD. In the oxytocin condition, the “congruency effect” (i.e., faster avoidance of facial anger and approach of facial happiness vice versa) was increased in both groups. Notably, patients with BPD exhibited a congruency effect toward angry faces in the oxytocin but not in the placebo condition.

**Conclusions:** This is the second report of deficient fast, automatic avoidance responses in terms of approach behavior toward interpersonal threat cues in patients with BPD. Intranasally administered oxytocin was found to strengthen avoidance behavior to social threat cues and, thus, to normalize fast action tendencies in BPD. Together with the previously reported oxytocinergic reduction of social threat hypersensitivity, these results suggest beneficial effects of oxytocin on interpersonal dysfunctioning in BPD.

## Introduction

Interpersonal dysregulation is a prominent and lasting symptom of patients with borderline personality disorder (BPD). Patients with BPD report more often about frequent negative interactions, less social integration, and poorer social support than do healthy individuals (1). Factors influencing such experiences could be symptoms such as fear of abandonment and impulsive behavior and also deficits in social cognition (e.g., empathy, cooperation, emotion recognition, and regulation) (2, 3). A related aspect is hypersensitivity to threatening information when processing emotional states of others (4). Patients with BPD tend to detect subtle signals of threat and to focus their attention on threatening interpersonal cues (4–6). Furthermore, faster initial saccades into the eyes—the most threatening part—of angry faces in patients with BPD suggest approach rather than avoidance behavior to interpersonal threat cues (7). In an experimental approach–avoidance task (AAT), anger-prone women with BPD reacted faster in approaching than avoiding angry—potentially threatening—faces than healthy women did (8). In such tasks, appetitive stimuli, such as happy faces, usually trigger approach behavior in healthy participants, while aversive or threatening stimuli, such as angry faces, trigger avoidance (9). Hence, healthy participants are faster when instructed to approach happy faces and to avoid angry faces than vice versa. This has been referred to as the “congruency effect”: affect-congruent behaviors (approach happy/avoid angry) can be performed faster than affect-incongruent (approach angry/avoid happy) behaviors, which require the individuals to override fast affect-congruent tendencies (10–12). Taken together, there is increasing evidence that interpersonal dysfunctioning is associated with threat hypersensitivity and deficient avoidance of interpersonal threat in BPD, which may be a major factor underlying the high prevalence of reactive aggression in BPD (13).

Interestingly, the neuropeptide oxytocin has been found to modulate interpersonal processes, such as threat sensitivity and avoidance in healthy individuals (14). There is some evidence from healthy samples, which suggests that oxytocin may influence social threat approach (15). For instance, increased approach behavior was found toward angry faces after intranasal oxytocin administration in healthy male participants with low levels of social anxiety (11). Approach behavior also increased toward pleasant social stimuli (e.g., pictures of attractive men) in the oxytocin condition compared with the placebo condition in healthy women (16). However, there is inconsistency in data since a study by Theodoridou et al. (17) did not find any effects of intranasal oxytocin on behavioral tendencies to facial and non-facial stimuli depicting one of five emotions, except for a general prolongation of reaction times, in a large sample of healthy men and women.

Recently, oxytocin has become a rising topic in BPD research and is currently tested as an adjuvant in the treatment of BPD (18). Although the number of studies investigating the effects of oxytocin in BPD is still small and results remain heterogeneous, the first beneficial effects of oxytocin on threat processing have been reported: First, the intranasal administration of oxytocin reduced BPD patients' attention bias to angry faces in a dot probe task (19). Second, the above-mentioned tendency for faster and more saccades toward the eyes of angry faces was not found in patients with BPD following intranasal oxytocin administration, suggesting a decrease of social threat hypersensitivity (7). Until now, oxytocinergic modulation of approach–avoidance behavior has not been studied in BPD.

Given this background, we investigated the effects of oxytocin on approach–avoidance behavior using an AAT with angry and happy faces in 53 women with BPD and 61 healthy women. In a randomized, double-blind design, participants received either 24 IU of oxytocin or placebo intranasally. We expected a replication of the results by Bertsch et al. (8) with more approach behavior to angry faces in BPD in comparison to avoidance behavior. In the oxytocin condition, we expected reduced approach behavior toward potentially threatening angry stimuli in patients with BPD.

## Materials and Methods

### Participants

Fifty-three unmedicated women with a current *Diagnostic and Statistical Manual of Mental Disorders*, fourth edition (DSM-IV), diagnosis of BPD (BPD; *M*_number of IPDE symptoms_ = 6.43, *SD* = 1.17, range: 5–9; *M*_age_ = 30.19, *SD* = 7.51 years, range: 19–49 years; 26 oxytocin/27 placebo) and 61 healthy female controls (HC; *M*_age_ = 28.36, *SD* = 7.65 years, range: 18–52 years; 30 oxytocin/31 placebo) with no lifetime psychiatric diagnosis took part in the study (Table 1). Originally, 60 patients and 62 HCs were assessed; however, six patients had to be excluded because they had <50% valid trials (correct joystick movement in accordance to task of condition) in one or more conditions of the paradigm, and one patient and one HC had to be excluded because of technical difficulties in the recording.

**Table 1:** Demographic, hormonal, and clinical characteristics.

	**BPD**		**HC**				
	**M**	**SD**	**M**	**SD**	***T*/*F*_**df**_**	***p***	**$\eta_{\text{p}}^{2}$**
Age (in years)	30.19	7.51	28.36	7.65	1.28_112_	0.202	
IQ	108.71	10.89	115.66	11.15	−3.34_111_	0.001^*^	
Progesterone (ng/ml)	2.80	3.84	1.25	2.14	2.70_112_	0.008	
Estradiol (pg/ml)	65.69	51.64	65.80	66.37	−0.10_112_	0.992	
BSL	1.50	0.86	0.11	0.14	122.39_1,104_	<0.001^*^	0.54
BDI	21.23	11.22	1.76	2.43	142.04_1,104_	<0.001^*^	0.58
ECR-R anxiety	5.12	1.11	2.06	0.85	253.22_1,110_	<0.001^*^	0.70
ECR-R avoidance	4.01	1.13	2.79	0.64	152.74_1,110_	<0.001^*^	0.58
DERS	132.43	19.68	65.30	12.49	417.17_1,105_	<0.001^*^	0.80
BIS	89.33	13.28	59.18	10.18	151.62_1,106_	<0.001^*^	0.59
STAXI: trait anger	27.67	6.13	17.23	3.93	92.92_1,105_	<0.001^*^	0.47
CTQ	60.72	23.39	30.63	7.85	64.50_1,105_	<0.001	0.39

*M, means; SD, standard deviation. Significant p-values marked with an asterisk. Bonferroni corrected for multiple testing. Factor “IQ” included as a covariate for questionnaire data. BSL, Borderline Symptom List; BDI, Beck Depression Inventory; ECR-R, Experiences in Close Relationships-Revised; DERS, Difficulties in Emotion Regulation Scale; BIS, Barratt Impulsiveness Scale; STAXI, State–Trait Anger Inventory; CTQ, Childhood Trauma Questionnaire*.

Exclusion criteria were a current and lifetime diagnosis of bipolar disorder, schizophrenia, schizoaffective disorder, and alcohol or drug (nicotine excluded) dependence over the last 12 months (assessed via urine toxicology screenings and interviews), pregnancy, severe medical illness, severe visual handicap, neurological disorders, and organic brain damage. The number of comorbidities can be seen in Table 2. Participants were recruited through a central unit for diagnostics, which is part of the Clinical Research Unit funded by the German Research Foundation (DFG; KFO 256) (20). Additionally, participants had to be free of psychotropic medication for at least 2 weeks before participation.

**Table 2:** Current and lifetime comorbidities in BPD.

**Comorbidity**	**Current (*n*)**	**Lifetime *(n)***
Mood disorder	15	45
Anxiety disorder	20	23
Obsessive–compulsive disorder	3	4
Posttraumatic stress disorder	9	21
Eating disorder	8	24
Substance dependence	0	9
ASPD	1	2
APD	19	20

*ASPD, antisocial personality disorder; APD, avoidant personality disorder*.

### Diagnostic Assessment

Axis I and II disorders were assessed by the Structured Clinical Interview (SCID-I) (21) and the International Personality Disorder Examination (IPDE) (22), respectively. Diagnoses were given by trained and qualified diagnosticians in accordance with DSM-IV (23). Intelligence (IQ) was estimated by the use of Raven's progressive matrices (24). Self-rating questionnaires assessed borderline symptom severity (Borderline Symptom List, BSL) (25), depressiveness (Beck Depression Inventory, BDI) (26), childhood traumatization (Childhood Trauma Questionnaire, CTQ) (27), attachment (Experiences in Close Relationships-Revised, ECR-R) (28), emotion dysregulation (Difficulties in Emotion Regulation Scale, DERS) (29), impulsivity (Barratt Impulsiveness Scale, BIS) (30), and trait anger (State–Trait Expression Inventory, STAXI) (31).

### Hormonal Assessment

A blood sample was taken in 5-ml heparin-plasma Vacutainer tubes in order to analyze progesterone and estradiol to control for menstrual cycle. Samples were analyzed at the Central Laboratory of the University of Heidelberg, Germany, using chemiluminescence immunoassays (ACS:180® Estradiol-6 II test from Bayer Diagnostics, Germany). The assay detection limits were 0.2 ng/ml for progesterone and 11.8 pg/ml for estradiol. There was a minimal cross-reactivity with other related compounds. For progesterone, the coefficient for intra-assay precision was <3%, and the coefficients of variation for inter-assay and intra-assay precision were <6%. For estradiol, the coefficient for intra-assay precision was <6%, and the coefficients of variation for inter-assay and intra-assay precision were <7%.

### Approach–Avoidance Task

The AAT (32) consisted of 192 trials in four blocks with 16 training trials and 32 main trials per block. The intertrial interval was 2–4 s, and between blocks laid 21–24 s. Blocks were counterbalanced across participants. Happy and angry faces with direct gaze from eight actors [four male and four female; selected from (33)] were presented as stimuli in a pseudorandomized order. Each stimulus was presented twice per block during the main trials and 12 times in total. Before each block, participants received either the instruction to push angry faces away from them and pull happy faces toward them (congruent condition) or the opposite instruction (incongruent condition) using a joystick (Attack 3, Logitech, Apples, Switzerland). Pushing or pulling the joystick resulted in shrinking or enlarging of the face (“zooming effect”) (32). Then participants had to move the joystick back to the starting position. Participants were instructed to react as fast as possible. All participants underwent both conditions. The number of correct trials and reaction times, that is, the time from stimulus presentation until completion of the movement of the joystick, were recorded.

### Experimental Protocol

The study was conducted with a double-blind, placebo-controlled design. Participants were screened via telephone and participated in a face-to-face diagnostic interview prior to the experiment. Experiments took place in the afternoon between 12 and 5 p.m. in order to control for diurnal hormonal patterns at the University Hospital of Heidelberg. Participants were asked to abstain from caffeine intake and smoking on the experimental day and from food intake 2 h before the experiment. Each participant was informed about the study protocol, gave written informed consent, and provided a urine sample for drug screening and pregnancy test as well as a blood sample for hormonal assessments. Then participants filled out questionnaires. Following a protocol of our previous studies (7, 34–36), oxytocin (24 IU, Syntocinon Spray, Novartis, Basel, Switzerland) or placebo (spray with the same inactive ingredients but oxytocin) was intranasally applied by the participant with six puffs of 2 IU in each nostril. After administration, participants were asked to lie back in a 45° angle for 10 min. The drugs were prepared by an independent pharmacist according to an externally computerized randomization list (simple randomization). Electrodes for EEG measurements in another experiment were applied, and participants performed an emotion classification paradigm prior to the here reported experiment (results will be published elsewhere). Seventy-five minutes after application, participants were seated in front of a laptop with an attached joystick in a dimly lit, sound-attenuated room. Participants were instructed and completed a short training session. The duration of the AAT was ~12 min.

### Ethical Standards

The study was conducted according to the ethical standards of the relevant national and institutional committees on human experimentation and with the Helsinki Declaration of 1975, as revised in 2008. It was approved by the Ethics Committee of the Medical Faculty at Heidelberg University, Germany. All participants gave written informed consent and received equal monetary compensation for their participation.

## Data Analysis

Data processing was performed in R (37) and data analyses in IBM SPSS statistics 25 (IBM, Armonk, NY). Independent *t*-tests were used to analyze differences in age, intelligence, and hormonal data between patients and HCs. Analyses of covariance (ANCOVAs) were performed for questionnaire data controlling for IQ due to a significant group difference. Bonferroni correction was used to control for multiple comparisons.

For AAT data, trials with reaction times of <150 or >1,500 ms were excluded from further analysis (included trials in analysis: 95.5%) (32). Participants with <50% valid trials (correct joystick movement in accordance to task of condition) in one or more conditions were excluded (*n* = 6) (38). Initial reaction time, that is, time from stimulus presentation until movement onset, was used for analysis. To analyze behavioral data, 2 × 2 × 2 × 2 repeated-measure analyses of covariance (rm-ANCOVA) with group (BPD and HC) and substance (oxytocin and placebo) as between-subjects factors and emotion (angry and happy) and congruency (congruent and incongruent) as within-subjects factors were used. IQ and estradiol and progesterone levels in order to control for hormonal levels and menstrual cycle were included as covariates. Dunn's multiple comparisons with Bonferroni correction for multiple testing were calculated as *post hoc* tests. Results were considered to be significant at *p* < 0.05. Partial eta squared ($\eta_{\text{p}}^{2}$) was used as a measure of effect sizes for rm-ANCOVAs and Cohen's *d* as a measure of effect sizes for *post-hoc* tests.

In an exploratory approach, correlations were calculated to test for possible associations between the congruency effect in angry faces (incongruent–congruent) and borderline symptom severity (IPDE criteria), attachment (ECR-R), impulsivity (BIS), trait anger (STAXI), or emotion dysregulation (DERS) separately in the oxytocin and placebo conditions in patients with BPD. Pearson's correlations were used for normally distributed data, and Spearman's correlation was used for skewed data (IPDE criteria only).

## Results

### Demographics and Clinical Scores

The groups did not differ with regard to age, but a significant difference was found in the IQ; that is, patients with BPD had a lower—but still in the normal range—IQ than did HC. Groups differed significantly in all questionnaire data (see Table 1 for detailed information).

### Approach–Avoidance Behavior

**Figure 1:**
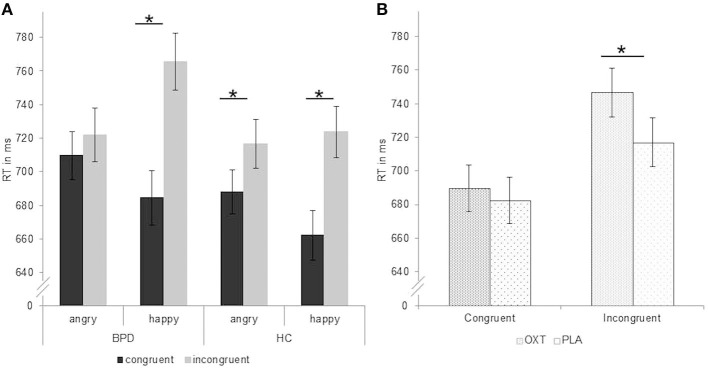
Reaction times in ms (mean ± standard error) during performance of the approach–avoidance task. **(A)** Significant group-by-emotion-by-congruency interaction with missing congruency effect for angry faces in patients with borderline personality disorder (BPD). **(B)** Significant substance by congruency interaction with longer reaction times after application of oxytocin than placebo in the incongruent condition over all participants. Factor “IQ” and estradiol and progesterone levels included as covariates. Significant comparisons are marked with an asterisk indicating *p* < 0.05 at the *post-hoc* test. OXT, oxytocin; PLA, placebo.

There was a significant group-by-emotion interaction [*F*_(1,106)_ = 6.24, *p* = 0.014, $\eta_{\text{p}}^{2}$ = 0.06; Table 3] with faster reaction times in patients with BPD for angry than happy faces (*p* < 0.05, *d* = −0.09) and faster reaction times in HC for happy than angry faces (*p* < 0.05, *d* = 0.09). Furthermore, the analysis also revealed a significant group-by-emotion-by-congruency interaction [*F*_(1,106)_ = 5.36, *p* = 0.022, $\eta_{\text{p}}^{2}$ = 0.05; Figure 1]. *Post-hoc* tests showed that, in patients with BPD, reaction times for angry faces did not differ between congruent (avoid) and incongruent (approach) conditions (*p* > 0.05, *d* = −0.11), while HC responded significantly slower in the incongruent (approach angry) than congruent (avoid angry) condition (*p* < 0.01, *d* = −0.26), which is consistent with the congruency effect. For happy faces, both groups showed slower reactions in the incongruent (approach happy) than congruent (avoid happy) condition (BPD: *p* < 0.01, *d* = −0.68; HC: *p* < 0.01, *d* = −0.52).

**Table 3:** Mean reaction times (M) in ms and standard error (SE) to angry and happy faces in patients with borderline personality disorder (BPD) and healthy controls.

	**BPD**		**HC**	
	**M**	**SE**	**M**	**SE**
Angry	715.73	14.24	702.29	13.06
Happy	725.07	15.73	692.89	14.43

*Factor “IQ” and estradiol and progesterone levels included as covariates*.

We found a significant substance-by-congruency interaction [*F*_(1,106)_ =4.18, *p* = 0.043, $\eta_{\text{p}}^{2}$ = 0.04; Figure 1]. *Post-hoc* tests revealed slower reaction times for incongruent trials in the oxytocin than in the placebo condition (*p* < 0.01, *d* = 0.27), while no substance effect emerged for congruent trials (*p* > 0.05, *d* = 0.52).

**Figure 2:**
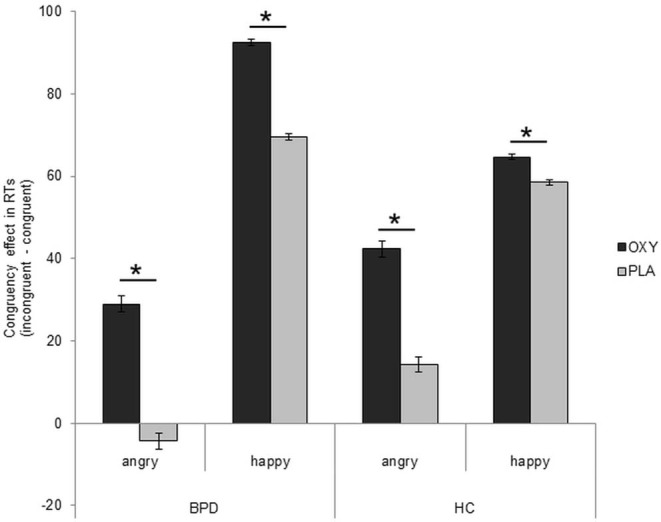
Presentation of congruency effect after application of oxytocin in patients with borderline personality disorder (BPD). Difference scores (incongruent–congruent conditions) of reaction times in ms (mean ± standard error). Factor “IQ” and estradiol and progesterone levels included as covariates. Significant comparisons are marked with an asterisk indicating *p* < 0.05. OXT, oxytocin; PLA, placebo.

There were no further significant main or interaction effects (all *F* ≤ 0.02, *p* ≥ 0.05, $\eta_{\text{p}}^{2}$ ≤ 0.01; Figure 2), and the correlation analyses did not reveal any significant associations with borderline symptom severity or self-report data in the oxytocin (IPDE: *r*_s_ = 0.29, *p*_s_ = 0.152; ECR-R anxiety: *r* = 0.10, *p* = 0.631; ECR-R: avoidance: *r* = −0.10, *p* = 0.630; BIS: *r* = −0.05, *p* = 0.795; STAXI: *r* = 0.19, *p* = 0.363; DERS: *r* = 0.05, *p* = 0.826) or placebo (IPDE: *r*_s_ = 0.02, *p*_s_ = 0.935; ECR-R anxiety: *r* = 0.13, *p* = 0.519; ECR-R: avoidance: *r* = −0.14, *p* = 0.489; BIS: *r* = 0.02, *p* = 0.920; STAXI: *r* = −0.23, *p* = 0.245; DERS: *r* = −0.27, *p* = 0.170) condition in patients with BPD.

## Discussion

The study revealed three major findings: First, patients with BPD responded faster to angry than happy faces, while healthy participants showed the opposite pattern, that is, faster responses to happy than to angry faces. Second, patients with BPD were as fast in approaching as in avoiding angry faces and did not show the typical congruency effect for angry faces. Third, reaction times in incongruent conditions (approach angry and avoid happy faces) were slower in the oxytocin condition across both groups, leading to a more pronounced congruency effect under oxytocin (Figure 2); in the case of patients with BPD, this prolongation resulted in a normalization of behavioral tendencies in response to angry faces in comparison to previous results (8); that is, they were faster in avoiding than in approaching angry faces.

Our first finding of faster reaction times to angry compared with happy faces is in line with the theory that patients with BPD show a bias toward threatening information (4). For example, patients with BPD show faster initial saccades into the eyes of angry faces, (7) are more likely to recognize even subtle signals of anger in facial stimuli (5), and misconstrue happy, fearful, or neutral faces more often as angry (6). In the healthy participants though, positive stimuli triggered faster emotional reactions than negative stimuli, replicating previous findings (39).

In line with our *a priori* hypothesis, our second finding replicated a missing congruency effect for angry faces in an independent sample of BPD patients (8). Patients with BPD were as fast in approaching as in avoiding angry faces, suggesting a deficit in fast avoidance tendencies for interpersonal threat cues. Notably, patients in the current sample were not faster in approaching than avoiding angry faces as reported by Bertsch et al. (8) who, however, only included anger-prone patients with BPD. Such anger-prone patients might feel particularly provoked by interpersonal threats and have more pronounced avoidance deficits compared to an “average” BPD sample as included in this study, increasing the risk of aggressive behavior. Since we did not find any significant correlations with anger or other trait measures in the current study and the heterogeneity among patients with BPD is large, further studies with larger groups are needed to further elucidate the circumstances under which deficient threat avoidance, that is, increased approach behavior toward threat stimuli, is related to anger outburst and aggressive behavior.

Finally, our third and most important finding confirms our hypothesis of an oxytocinergic modulation of approach–avoidance behavior in BPD. Across both groups, participants in the oxytocin condition responded generally slower than those in the placebo condition. This is consistent with previous reports of prolonged reaction times in the AAT after oxytocin administration (17, 39). Furthermore, according to a substance-by-congruency interaction, oxytocin particularly increased reaction times in affect-incongruent (approach angry and avoid happy faces), but not affect-congruent (avoid angry and approach happy faces) conditions. Most interestingly, with oxytocin administration, patients with BPD were faster in avoiding than approaching angry faces, thus showing the “normal” congruency effect. This oxytocin-induced normalization of approach–avoidance behavior in comparison to previous results (8) might be related to more cognitively controlled action tendencies to social threat cues as suggested by data indicating reduced prefrontal–amygdala communication during deficient emotional action control in terms of increased approach behavior toward angry faces in BPD in a functional neuroimaging study using the AAT (8). Oxytocin might also affect amygdala activation, a region involved in the processing of fast emotional behavioral tendencies since a previous neuroimaging study in healthy men has revealed decreased amygdala activation after oxytocin vs. placebo administration during threat approach, but not avoidance (39). Oxytocin effects on the amygdala were also observed in patients with BPD who showed not only less fast and less frequent saccades but also lower amygdala activity toward angry eyes compared to patients in the placebo group (7). Although we can only speculate about the neural underpinnings of the current effects, an oxytocinergic modulation of amygdala activation and/or prefrontal–amygdala coupling affecting cognitive control seems likely.

It needs to be noted that oxytocin had similar behavioral effects in patients and HCs and that no significant interaction with group was found. Our results also partly differ from those of previous studies where oxytocin had a reinforcing effect on approach behavior toward threatening stimuli in healthy volunteers (11, 15). The heterogeneity of oxytocinergic effects on behavioral tendencies in healthy individuals needs to be addressed in further studies and could be related to differences in sex or other sample characteristics (anxiety level and attachment style) as well as methodological issues, such as paradigm, design (within vs. between subject), or context (neuroimaging vs. behavioral lab) (15).

When the current findings are interpreted, several limitations need to be considered, such as the limited sample size, the between-subject design, and the comorbid mental disorders in the BPD group. Additionally, we specifically focused on a female sample in order to avoid potential bias induced by sex. However, we do not have reliable data on hormonal contraception of the participants, which could be a possible confounding factor. The AAT was conducted ~75 min after substance application, which is still in the range of elevated peripheral and presumably also central oxytocin levels (40, 41) but might be past its peak levels (30–60 min after application) in the cerebral spinal fluid (42). Therefore, a replication in a larger sample including male and female participants and a clinical control group, as well as including imaging techniques in order to understand more about underlying mechanisms, are necessary next steps. Additionally, dose-dependent effects of oxytocin need to be investigated in future studies, preferably in a pre–post design. If replication studies prove our results as reliable, future study designs need to extend to more naturalistic environments in order to examine oxytocin as a potential drug for BPD treatment.

Despite these potential shortcomings, this study revealed an oxytocin-induced normalization of threat avoidance behavior in patients with BPD by prolonging reaction times in affect-incongruent (approach angry and avoid happy faces) conditions. Together with previous results and consistent with a recently published review (3), the current findings suggest beneficial effects of oxytocin for patients with threat hypersensitivity and deficient threat avoidance, as found in BPD.

## Data Availability Statement

The datasets generated for this study will not be made publicly available Requests will need to be reviewed and agreed upon with the Clinical Research Unit (KFO 256) as the data was collected as part of it.

## Ethics Statement

The studies involving human participants were reviewed and approved by Ethics Committee of the Medical Faculty at Heidelberg University, Germany. The patients/participants provided their written informed consent to participate in this study.

## Author Contributions

IS: acquisition of data, analysis and interpretation of data, and drafting and finalizing of the manuscript. SB: acquisition of data, analysis and interpretation of data, and critical revision. IV and KR: study conception and design and critical revision. AS: acquisition of data and critical revision. SH: study conception and design, drafting of the manuscript, and critical revision. KB: study conception and design, analysis and interpretation of data, drafting of the manuscript, critical revision.

### Conflict of Interest

The authors declare that the research was conducted in the absence of any commercial or financial relationships that could be construed as a potential conflict of interest.
